# Behavioral phenotyping of a rat model of the BDNF Val66Met polymorphism reveals selective impairment of fear memory

**DOI:** 10.1038/s41398-022-01858-5

**Published:** 2022-03-07

**Authors:** Emily J. Jaehne, Jessica N. Kent, Emily J. Antolasic, Bradley J. Wright, Jereme G. Spiers, Kerstin C. Creutzberg, Federico De Rosa, Marco A. Riva, Caryl E. Sortwell, Timothy J. Collier, Maarten van den Buuse

**Affiliations:** 1 Department of Psychology and Counselling, School of Psychology and Public Health, La Trobe University, Melbourne, Australia; 2 Department of Biochemistry and Genetics, La Trobe Institute for Molecular Science, La Trobe University, Melbourne, Australia; 3 Department of Pharmacological and Biomolecular Sciences, University of Milan, Milan, Italy; 4 Biological Psychiatry Laboratory, IRCCS Istituto Centro San Giovanni di Dio Fatebenefratelli, Brescia, Italy; 5 Department of Translational Neuroscience, College of Human Medicine, Michigan State University, Grand Rapids, USA; 6 Hauenstein Neuroscience Center, Mercy Health Saint Mary’s, Grand Rapids, USA; 7 Department of Pharmacology, University of Melbourne, Melbourne, Australia; 8 College of Public Health, Medical and Veterinary Sciences, James Cook University, Townsville, Australia

**Keywords:** Neuroscience, Psychiatric disorders

## Abstract

The common brain-derived neurotrophic factor (BDNF) Val66Met polymorphism is associated with reduced activity-dependent BDNF release and increased risk for anxiety disorders and PTSD. Here we behaviorally phenotyped a novel Val66Met rat model with an equivalent valine to methionine substitution in the rat Bdnf gene (Val68Met). In a three-day fear conditioning protocol of fear learning and extinction, adult rats with the Met/Met genotype demonstrated impaired fear memory compared to Val/Met rats and Val/Val controls, with no genotype differences in fear learning or extinction. This deficit in fear memory occurred irrespective of the sex of the animals and was not seen in adolescence (4 weeks of age). There were no changes in open-field locomotor activity or anxiety measured in the elevated plus maze (EPM) nor in other types of memory measured using the novel-object recognition test or Y-maze. BDNF exon VI expression in the dorsal hippocampus was higher and BDNF protein level in the ventral hippocampus was lower in female Val/Met rats than female Val/Val rats, with no other genotype differences, including in total BDNF, BDNF long, or BDNF IV mRNA. These data suggest a specific role for the BDNF Met/Met genotype in fear memory in rats. Further studies are required to investigate gene–environment interactions in this novel animal model.

## Introduction

Brain-derived neurotrophic factor (BDNF) is expressed throughout the brain and has a primary role in neuronal development, survival, differentiation, and plasticity in both the developing and mature brain [1–4]. BDNF is implicated in the pathophysiology of stress-related mood disorders in particular [5, 6]. Reduced BDNF levels have been observed in depression patients and may contribute to reduced hippocampal volume and cognitive deficits seen in depression [7–9].

The Val66Met single nucleotide polymorphism (SNP) of the BDNF gene (rs6265) has been studied extensively in the context of psychiatric disorder susceptibility, given it results in diminished activity-dependent BDNF secretion and aberrant trophic function, and has been suggested as a putative locus of risk for anxiety and affective disorders [1, 2, 6]. A meta-analysis conducted by Frustaci et al. [10] found that individuals homozygous for the Met allele have an increased risk for developing generalized anxiety, although other association studies have found contrasting results when comparing the incidence in Val and Met carriers with different anxiety traits [11–15]. Further association studies have confirmed that the Met allele may be a risk allele for psychiatric disorders [16–18].

We have previously characterized the behavioral phenotype of a Val66Met mouse model. Met/Met mice were shown to display decreased contextual fear memory in a fear conditioning paradigm and also demonstrated impaired spatial memory in the Y-maze, with no differences in anxiety seen in the light-dark box [19]. Met/Met mice were also shown to have higher immobility in the forced swim test (FST) compared to mice with the Val/Val genotype [20]. In contrast, others showed increased anxiety-like behavior in Met/Met mice in comparison to Val/Val counterparts in the open field and elevated plus maze (EPM) paradigms [1]. Further to these findings, we have also investigated behavior in rats heterozygous (het) for the BDNF gene, with BDNF levels 50% of that in wildtype (WT) rats [21, 22]. BDNF het rats showed a slight decrease in freezing behavior during the fear learning phase of a fear conditioning protocol but did not show differences in fear memory or extinction. Other researchers have shown BDNF het rats to have impaired fear extinction memory [23, 24], while several studies have shown that infusion of BDNF into the brain, in particular, the infralimbic cortex and hippocampus, lead to impairments in the extinction of fear memory and other changes in the fear learning response [25–28]. Much of the current literature in both human and animal studies suggests that the Met allele is associated with deficits in the persistence of fear memories when compared to the Val/Val genotype [29–33]. BDNF het rats also showed some impairment in spatial memory in the Y-maze, but no differences compared to WT rats in the novel object recognition task (NORT) [22], as well as increased levels of anxiety in the open field but not the elevated plus maze (EPM), and also showed no differences in behavior in the FST [21]. These results suggest that BDNF is likely to play a role in learning and cognition, as well as possibly anxiety- and affective-type behaviors; however, this depends on the animal model used.

Recently, a rat model of the Val66Met polymorphism has been developed carrying a valine to methionine substitution (Val68Met) in the rat Bdnf gene [34]. While all previous animal models of the Val66Met polymorphism have been in mice due to the ability to more easily genetically modify the mouse genome previously, rats have been the animal model of choice for behavioral neuroscience research for over a century [35, 36]. This is primarily due to the rich and complex behavioral repertoire of the rat compared to mice [36]. Following the release of the rat genome [37], a shift back to the rat in behavioral neuroscience studies is being observed presently. Here we have used this novel model to extend our previous findings on affective and emotional behaviors in Val66Met mice and BDNF het rats. Because rats have two additional threonine amino acids at positions 57 and 58, this rat Val68Met is equivalent to the human Val66Met SNP. In vitro BDNF release was confirmed to be reduced from cultured neurons from Met/Met rats without altering total BDNF brain tissue content [34]. The current study investigates the baseline behavioral phenotype of these Val68Met rats over a range of anxiety and memory tasks, with a particular focus on fear behavior which BDNF Val66Met has been implicated in [6]. For this, we specifically focused on fear learning, memory, and extinction in both adult and adolescent Val68Met rats, as several aspects of fear conditioning show marked differences between these developmental stages [38–40].

The BDNF val66met polymorphism changes transcriptional levels of *Bdnf* in rodents. The BDNF gene has a complex structure, containing multiple promoters that are responsible for the differential regulation of its transcription [3, 41–43]. Some BDNF mRNA variants are spatially segregated in subcellular compartments, with BDNF IV being predominant in proximal dendrites and BDNF VI in distal dendrites [44]. Furthermore, the 3′-coding exon has two different polyadenylation sites giving rise to two pools of BDNF transcripts, one with short and one with long 3′-UTR, the latter being responsible for targeting BDNF transcripts to dendrites [45]. As previous work has shown that Met/Met mice show altered expression of BDNF transcripts [46, 47], we assessed BDNF gene and protein expression in the Val68Met rats.

The results show significantly lower fear memory in adult Met/Met rats in the absence of other changes in anxiety, cognitive or developmental deficits. Subtle changes in BDNF gene expression and BDNF protein levels were found in the dorsal and ventral hippocampus, respectively, but these changes did not correlate with the deficit in fear memory.

## Methods

### Animals

Male and female outbred rs6265 (Val68Met) rats with the Met/Met genotype were originally obtained from Dr Caryl Sortwell (Michigan State University, MI, USA) and a breeding colony was established at the Australian Resource Center (ARC, WA, Australia). Full details of the generation of this rat model have been published [34]. Briefly, these rats carry the valine to methionine polymorphism (Val68Met) in the rat Bdnf gene (GenBank: NM_001270630; Ensembl: ENSRNOG00000047466). This Bdnf knock-in rat model was generated in Sprague-Dawley rats by Cyagen Biosciences (Santa Clara, CA, USA) using CRISPR/Cas-mediated homologous recombination [34]. For the present study, Met/Met founder rats at ARC were mated with Sprague-Dawley controls to generate heterozygous Val/Met rats. These animals were shipped to the La Trobe University Animal Research and Training Facility (LARTF) where they were used to produce offspring of all genotypes (Val/Val, Val/Met, Met/Met) for experimentation. In all cases, genotyping was done by Transnetyx (Cordova, TN, USA). Genotypes of the offspring showed the expected approximately 1:2:1 Mendelian distribution (Supplementary Table 1).

A total of 287 offspring of all three genotypes and both sexes were used in this study. Rats were housed at the LARTF 2–4/cage in individually-ventilated cages (Tecniplast, Buguggiate, Italy) with ad libitum access to rodent chow and tap water, and kept in a temperature (21 °C ± 2 °C) and humidity (55% ± 15%) controlled environment on a 12 h light cycle (lights on 0700 h; light inside the cage 12–22 lux). All behavioral testing was conducted between 8 am and 4 pm. All procedures were compliant with guidelines of the Australian Code of Practice for the Care and Use of Animals for Scientific Purposes set by the National Health and Medical Research Council of Australia and were approved by the La Trobe University Animal Experimentation Ethics Committee.

### Behavioral testing

Experiments started when rats were 7–8 weeks of age (53.4 ± 0.3 days of age). There were no differences in body weights between genotypes across the course of the experiment (Supplementary Fig. 1). Three separate cohorts of rats were used, with a different battery of tests conducted in each cohort. Rats of approximately equal numbers of each genotype and sex were randomly assigned to each testing cohort (see Supplementary Table 2 for exact group numbers), with no more than 2 of each genotype and sex from a single litter used/group. Cohort 1 underwent fear conditioning (day 1–3) and elevated plus maze (EPM; day 8). Cohort 2 underwent Y-maze (day 1) and novel object recognition test (NORT) including open field habituation (day 5–7). Cohort 3 underwent fear conditioning (day 1–3), EPM (day 6) and open field (day 9). Although an attempt was made to perform less stressful and shorter duration tests first with at least 3 days between each test for rats to recover from any possible disruptions, as previously used for behavioral batteries [22, 48, 49], fear conditioning in cohort 1 and 3 was conducted first due to the size of male rats inside the chambers. Experimenters were blinded to genotypes of rats during all behavioral testing.

A further cohort of rats was tested from 4 weeks of age (28.2 ± 0.2 days of age; Supplementary Table 2) to investigate whether any changes seen in adult rats were already apparent during early adolescence. These rats only underwent fear conditioning (day 1–3), EPM (day 6) and open field (day 9).

### Fear conditioning

All animals were trained and tested in fear conditioning chambers (Med Associates, St Albans, VT, USA). Motion and freezing of the animals were recorded using automated near-infrared video tracking software (VideoFreeze, Med Associates). Two different contexts were used, context A and B. Context A consisted of aluminum walls, no house light, and grid floors with stainless steel rods which were cleaned with a water and peppermint essence solution in-between trials. Context B consisted of white acrylic walls, with house light on, sawdust bedding underneath the grid floor (identical to context A) and were cleaned with water in-between trials.

On Day 1 (fear learning acquisition) the animals were placed in either context A or B. Approximately even numbers of all groups were placed in each context to prevent the influence of differences in freezing behavior between the contexts. Following a 3 min habituation period, the animals were exposed to three 30-s 80 dB SPL tones as the conditioned stimulus (CS) paired with a one-second foot shock (0.7 mA) as the unconditioned stimulus (US) administered through the metal grid on the floor of the chambers. Each CS-US pairing was separated with a three-minute interval with a total session duration of 11 min. On Day 2 (fear memory and extinction learning) rats were placed inside the opposite context to Day 1. Following a 3 min habituation period, they were exposed to 40 CS tones with no shock, separated by five-second intervals for a total session duration of 27 min. This same procedure was repeated on Day 3 (extinction memory) [22].

Percentage of time spent freezing in each component was calculated by the VideoFreeze software by dividing the amount of time the animal spent motionless by the total amount of time spent within that component. Testing components were defined as ‘baseline’ where the period was without a tone or ‘testing’ where the period was with a tone. Testing component data from Day 2 and 3 were averaged in groups of 10 CS periods.

### Elevated plus maze

The EPM test took place on a platform elevated 50 cm from the floor with four arms 50 cm in length and 10 cm in width, resembling the shape of a plus sign. Two of the arms were enclosed with walls that were 50 cm in height, while the remaining two arms did not have walls. The center of the EPM, a 10 cm × 10 cm area, was open to allow rats to move freely between the arms. Each rat was placed in the center of the platform and was left to explore for 5 min [21, 50, 51]. The rats’ movements were recorded by video camera and analyzed with Ethovision software (Noldus) during this time, and the number of entries and time spent in each arm were recorded. Rats tend to show an aversion toward the open arms, presumably due to innate fear or anxiety. Therefore, more time spent in the open arms is considered to indicate lower levels of anxiety.

### Open field and novel object recognition test

On two consecutive days prior to NORT, the rats were habituated to the open field arena. Data from Day 1 of this habituation were used as the open field data. The arena consisted of a large enclosure, approximately 100 × 100 cm, enclosed with walls 50 cm in height. Each rat was placed at the same location close to a wall in the open field and left to explore for 10 min. The rats’ movements during this time were recorded by video camera and analyzed with Ethovision software (Noldus). Total distance traveled and time spent in a pre-set inner zone (60 × 60 cm) was recorded [21]. Rats show a preference for the periphery of the open field, and generally walk close to the walls. More time spent in the center zone of the open field is considered to indicate lower levels of anxiety.

On Day 3, two identical objects were placed near the right and left corners of one side of the arena. The rat was then placed in the arena facing the opposite wall and was allowed to explore the objects for 10 min (training phase; supplementary Fig. 4). Two hours later, the rat was placed back into the arena which now contained one familiar object from the training phase and one novel object for 5 min (testing phase) [22]. Objects used and side of the novel and familiar objects were randomized between all rats. Between all trials, the arena and objects were wiped down with 80 % ethanol to eliminate odor-based cues.

### Y-maze

The Y-maze was a Y-shaped apparatus with three arms (start arm and two test arms), each 50 cm long and 15 cm wide with walls 30 cm high. The arms were at a 120° angle from each other. The two test arms had different black and white symbols on either end wall. The maze floor was covered with sawdust and cage bedding which was mixed between trials to reduce olfactory cues. Behavior was tracked using Ethovision (Noldus) which measured time spent in each arm. Testing was conducted according to previously published protocols [19, 22, 48]. Rats were placed in the start arm of the Y-maze for two separate sessions. During the training phase, rats were allowed to explore the maze for 10 min with one of the test arms blocked off. Two hours later they were placed back in the start arm and allowed to explore the whole Y-maze for 5 min with all three arms open. The localization of novel and familiar arms was randomized between rats. Time spent in the novel test arm compared to the other arms (familiar and start) during the retention phase was used as a measure of short-term spatial recognition memory.

### Brain analysis

Two-three days following the final behavioral tests, rats were deeply anesthetized with CO_2_ and decapitated for collection of brain samples. Brains were rapidly frozen on dry ice and stored at −80 °C. A random selection of brains from cohort 3 were weighed (male Val/Val *n* = 10, male Val/Met *n* = 7, male Met/Met *n* = 13, female Val/Val *n* = 8, female Val/Met *n* = 11, female Met/Met *n* = 7). There were no differences between genotypes in this measure (Supplementary Fig. 2). Other rats (*n* = 6–8/group) had dorsal hippocampus dissected for gene expression analysis and ventral hippocampus dissected for BDNF ELISA.

### RNA extraction and gene expression analysis

Total RNA was isolated from dorsal hippocampus using PureZol RNA isolation reagent (Bio-Rad Laboratories) according to the manufacturer’s protocol. RNA concentration was measured using a NanoDrop spectrophotometer (Thermo Fisher) and RNA was subsequently used for quantitative real-time polymerase chain reaction (qRT-PCR) (CFX384 Real-Time system, Bio-Rad Laboratories). Samples were run in triplicate and β-actin was used as housekeeping gene. Primers for Bdnf long (Rn02531967_s1) and isoforms IV (Rn01484927_m1) and VI (Rn01484928_m1) were purchased from Thermo Fisher Scientific while β-actin (Fwd: CACTTTCTACAATGAGCTGCG, Rev: CTGGATGGCTACGTACATGG, probe: TCTGGGTCATCTTTTCACGGTTGGC) and Bdnf total (Fwd: AAGTCTGCATTACATTCCTCGA, Rev: GTTTTCTGAAAGAGGGACAGTTTAT, probe: TGTGGTTTGTTGCCGTTGCCAAG) primers and probes from Eurofins Genomics. All analyses were performed following the ΔΔCT method with β-actin as the endogenous control [52]. β-actin did not show any significant variability between the experimental groups. Data are presented as % compared to the male Val/Val group (set at 100%).

### BDNF ELISA

Hippocampal BDNF concentrations in the ventral hippocampus were determined using a commercially available sandwich ELISA (Biosensis Cat#: BEK-2211) according to the manufacturer’s instructions. To remove BDNF binding proteins and receptors, tissue lysates were prepared using the acid extraction method developed by [53] described in Appendix B of the manufacturer’s protocol. Briefly, samples were homogenized in 10 volumes of acidified lysis buffer and centrifuged at 15,000 × *g* for 30 min at 4 °C. Protein concentrations were determined in sample supernatants, and these were neutralized prior to ELISA. Sample concentrations were determined from a BDNF standard curve, corrected for dilution and protein concentration, and expressed as ng/mg protein. The average intra-assay and inter-assay coefficients of variation were 3.52% and 3.92%, respectively.

### Data analysis

Data were analyzed using SPSS statistical package (version 27) and Graph Pad Prism (version 9). Power analysis was not conducted to determine group numbers, however numbers chosen were based on previous studies using the same behavioral [22, 42, 48] and brain analyses [52]. The data were first checked for multivariate outliers using z-scores, and scores falling outside *z* = ±3.29 were removed. Additionally, some rats were excluded from NORT analysis due to low interaction with objects (0 s with one or both objects). The data were then checked for normality violations using skewness and kurtosis z-scores and scores outside *z* = ±1.96 considered to be violating normality. ANOVA has been suggested to be robust to mild violations of normality given that the sample size is at least 30 [54]. The homogeneity of variance assumption was tested using Levene’s test and if both this assumption and normality were violated, square root data transformation was conducted to transform the data closer to a normal distribution and non-violation of homogeneity (Fear conditioning Day 1, fear conditioning Day 3, Open field time in center, EPM time in open arms, NORT interaction time). In these cases, raw data is represented in all figures. The sphericity assumption of repeated measures was examined using Mauchly’s test, and upon violation, the Greenhouse-Geisser’s degrees of freedom adjustment was applied to the critical *p*-values.

Between-group differences were analyzed using a factorial Analysis of Variance (ANOVA), with repeated measures where appropriate. Between-subject factors were genotype (Val/Val, Val/Met, Met/Met) and sex (male, female). For fear conditioning, EPM and open field cohort was also included as a between-subjects factor. Analysis revealed no effect of cohort on either genotype or sex in any of these behaviors therefore full results will not be presented for this analysis. Where appropriate, post hoc analysis using Tukey’s multiple comparison was used to investigate significant main effects and interactions. To assess if freezing behavior was a sensitive predictor of genotype the area under the curve (AUC) from a receiver operator characteristic (ROC) curve analysis was calculated. The level of significance was set at *p* < 0.05 for all statistical analyses. As a measure of effect size, partial eta squared (ηp²) values were used, with cut-offs being ≥ 0.01 small, ≥ 0.06 medium, and ≥ 0.14 large (SPSS).

## Results

### No effect of genotype on growth and development

Body weight data showed the expected increase over the course of the experiment with males having significantly higher body weights than females (Supplementary Fig. 1). There were no differences between the genotypes in terms of body weight gain during post-weaning development or body weights at the time of behavioral testing (Supplementary Fig. 1). At the end of the experiments, a random selection of the rats showed that brain weight was not different between the genotypes either (Supplementary Fig. 2).

### Met/Met rats show decreased fear memory but no other differences in fear learning or extinction in a fear conditioning task

**Fig. 1:**
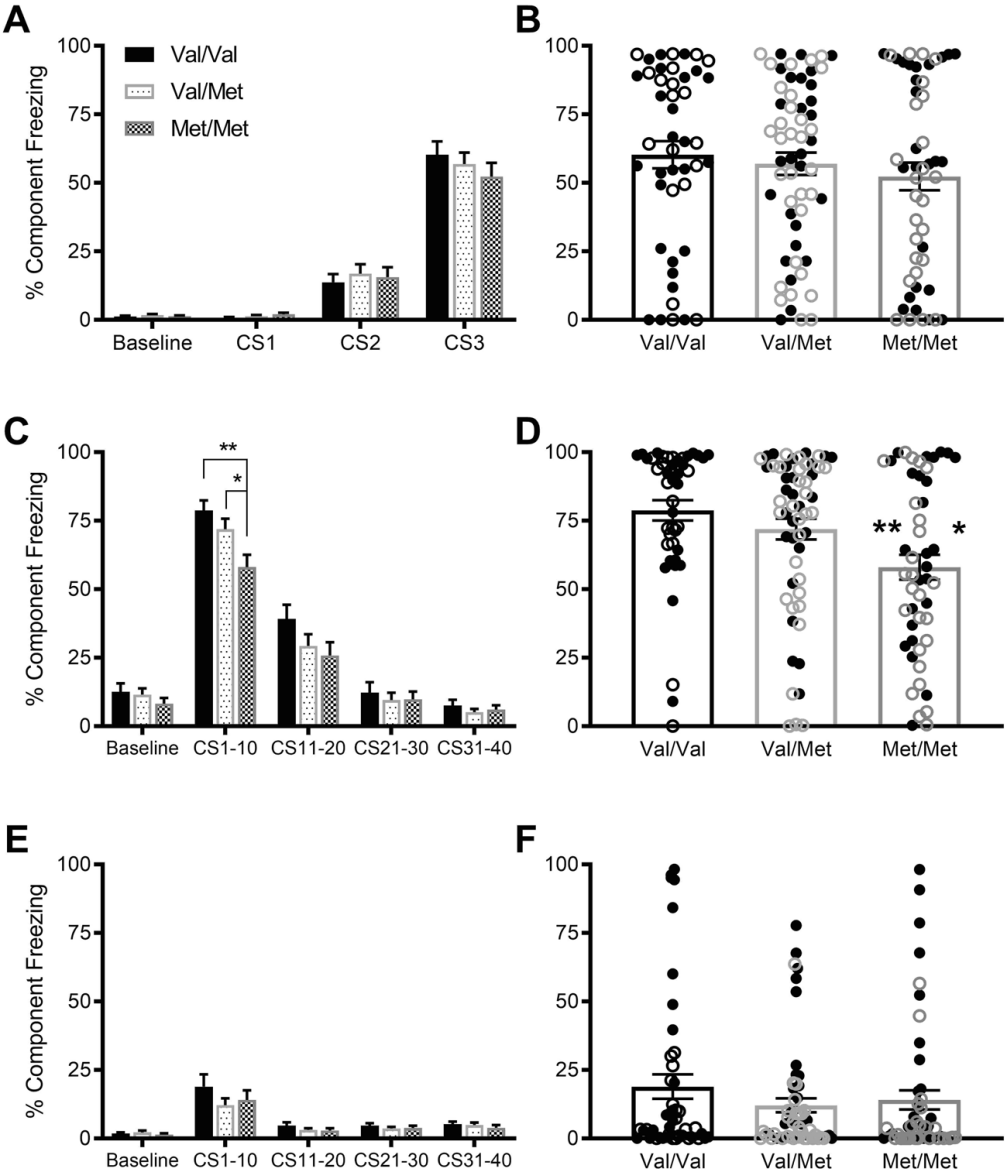
Fear conditioning in adult rats. Fear learning in adult rats across three CS tone periods on Day 1 (**A**) and CS3 only (**B**) shows no difference between genotypes. While there was no difference between genotypes in extinction learning as shown by the decrease in freezing across four periods of 10 CS tones on Day 2 (**C**), fear memory was shown to be decreased in Met/Met rats during CS1–10 (**D**). There were also no genotype differences in extinction memory on Day 3 when looking at average freezing across four periods of 10 CS tones (**E**) or when comparing freezing for CS1–10 only (**F**). Data are presented as mean ± SEM. Individual data points are only shown for (**B**), (**D**), and (**F**) for clarity. There were no significant interactions with sex therefore all data are presented as sexes combined (males closed symbols, females open symbols). **p < 0.001 vs. Val/Val, *p < 0.05 vs. Val/Met.

Analysis of the three CS periods on Day 1 (Fig. 1A; Fig. 1B for CS3 only) of the fear conditioning protocol showed a large main effect of CS (*F*(2,282) = 310.5, *p* < 0.001, ηp^2^ = 0.69), confirming fear learning, with freezing increasing from CS1 to CS3. There were no main effects of either sex or genotype on fear learning, and no significant interactions between CS and sex (*F*(2,282) = 0.071, *p* = 0.93, ηp^2^ = 0.001), CS and genotype (*F*(4,282) = 0.95, *p* = 0.45, ηp^2^ = 0.013) or CS, sex and genotype (*F*(4,282) = 0.34, *p* = 0.85, ηp^2^ < 0.001), suggesting similar levels of freezing and fear learning in all rats during this session.

Analysis of fear extinction on Day 2 across four groups of 10 CS tones (Fig. 1C) revealed a large main effect of CS (*F*(3,423) = 356.5, *p* < 0.001, ηp^2^ = 0.72), showing extinction learning, with freezing decreasing from CS1–10 to CS31–40. There were also small CS × sex (*F*(3,423) = 3.54, *p* = 0.025, ηp^2^ = 0.024) and a CS × genotype interactions (*F*(6,423) = 2.90, *p* = 0.018, ηp^2^ = 0.040), although not a significant CS × sex × genotype interaction (*F*(6,423) = 0.72, *p* = 0.60, ηp^2^ = 0.010). A medium main effect of sex (*F*(1,141) = 9.72, *p* = 0.002, ηp^2^ = 0.064) also showed that female rats freeze less than males during this session.

The significant interactions with CS were further explored by analyzing each group of 10 CS tones separately. In this analysis, CS1–10 was also used as a measure of fear memory, before the response starts to be extinguished (Fig. 1D). During this CS block, there was a medium effect of genotype (*F*(2,141) = 6.15, *p* = 0.003, ηp^2^ = 0.080) but not sex (*F*(1,141) = 2.22, *p* = 0.139, ηp^2^ = 0.015) or sex × genotype (*F*(2,141) = 0.71, *p* = 0.45, ηp^2^ = 0.010), suggesting an effect of the Val68Met genotype on fear memory not dependent on sex. Tukey’s multiple comparison post hoc test showed that Met/Met rats had lower freezing behavior than both Val/Val (*p* = 0.0018) and Val/Met rats (*p* = 0.036), indicating impaired fear memory. This genotype difference was no longer apparent during CS11–20 (*F*(2,141) = 1.29, *p* = 0.28, ηp^2^ = 0.018), CS21–30 (*F*(2,141) = 0.19, *p* = 0.83, ηp^2^ = 0.003) or CS31–40 (*F*(2,141) = 0.30, *p* = 0.74, ηp^2^ = 0.004) as the freezing response was extinguished in all rats, suggesting extinction learning was not affected by Val68Met genotype.

To further analyze the genotype differences in fear memory (CS1–10), we used Receiver Operator Characteristic (ROC) curve analysis (Supplementary Fig. 3). We calculated the AUC for each of the comparisons of freezing behavior between genotypes (Val/Val vs. Val/Met, Val/Val vs. Met/Met, Val/Met vs. Met/Met) for both fear learning and fear memory (Supplementary Fig. 3). The analysis revealed that freezing behavior during the fear learning component of the protocol on Day 1 was not a strong predictor of genotype (CS3; all *p* > 0.05). However, in the fear memory component on Day 2 (CS1–10), the comparison of the AUC revealed that higher levels of freezing behavior distinguished the Val/Val from the Met/Met genotype (AUC = 0.69, *p* = 0.001). Specifically, the ROC curve (Supplementary Fig. 3, B1) specifies that freezing behavior greater than 56% in the fear memory task distinguishes Val/Val rats from Met/Met rats. The 56% cut-off correctly identifies 91% (41/45) of Val/Val rats, whereas only 52% (26/50) of Met/Met rats were above this cut-off. After Bonferroni adjustment, the Val/Val to Val/Met (*p* = 0.171) and Val/Met to Met/Met (*p* = 0.036) comparisons were non-significant.

Analysis of the four groups of 10 CS tones on Day 3 (Fig. 1E) again shows a large main effect of CS (F(3,423) = 28.0, *p* < 0.001, ηp^2^ = 0.17). There were again a medium CS × sex interaction (*F*(3,423) = 8.46, *p* = 0.001, ηp^2^ = 0.057) and small main effect of sex (*F*(1,141) = 5.00, *p* = 0.027, ηp^2^ = 0.016), with females freezing less than males. There was no longer a CS × genotype interaction (*F*(6,423) = 0.092, *p* = 0.96, ηp^2^ = 0.001) and again no CS × sex × genotype interaction (*F*(6,423) = 0.56, *p* = 0.64, ηp^2^ = 0.008), suggesting extinction memory was similar in all genotypes. Extinction recall was further assessed by analyzing only the freezing for CS1–10 (Fig. 1F). There was a medium main effect of sex (*F*(1,141) = 11.3, *p* = 0.001, ηp^2^ = 0.074), with males freezing more than females, but not genotype (*F*(2,141) = 0.25, *p* = 0.78, ηp^2^ = 0.003) or genotype × sex (*F*(2,141) = 0.28, *p* = 0.75, ηp^2^ = 0.004) suggesting there is no effect of Val68Met genotype on extinction recall.

Comparison of the average of all 40 tones on Day 2 and Day 3 (data not shown) showed a large effect of day (*F*(1,141) = 333.7, *p* < 0.001, ηp^2^ = 0.70), indicating good extinction memory. There was again a large day × sex interaction (*F*(1,141) = 5.46, *p* = 0.021, ηp^2^ = 0.37) and a medium main effect of sex (*F*(1,141) = 11.2, *p* = 0.001, ηp^2^ = 0.074), with females freezing less than males. There were no significant day × genotype (*F*(2,141) = 3.03, *p* = 0.051, ηp^2^ = 0.041) or day × sex × genotype interactions (*F*(2,141) = 0.19, *p* = 0.83, ηp^2^ = 0.003), suggesting extinction memory was similar in all genotypes.

**Fig. 2:**
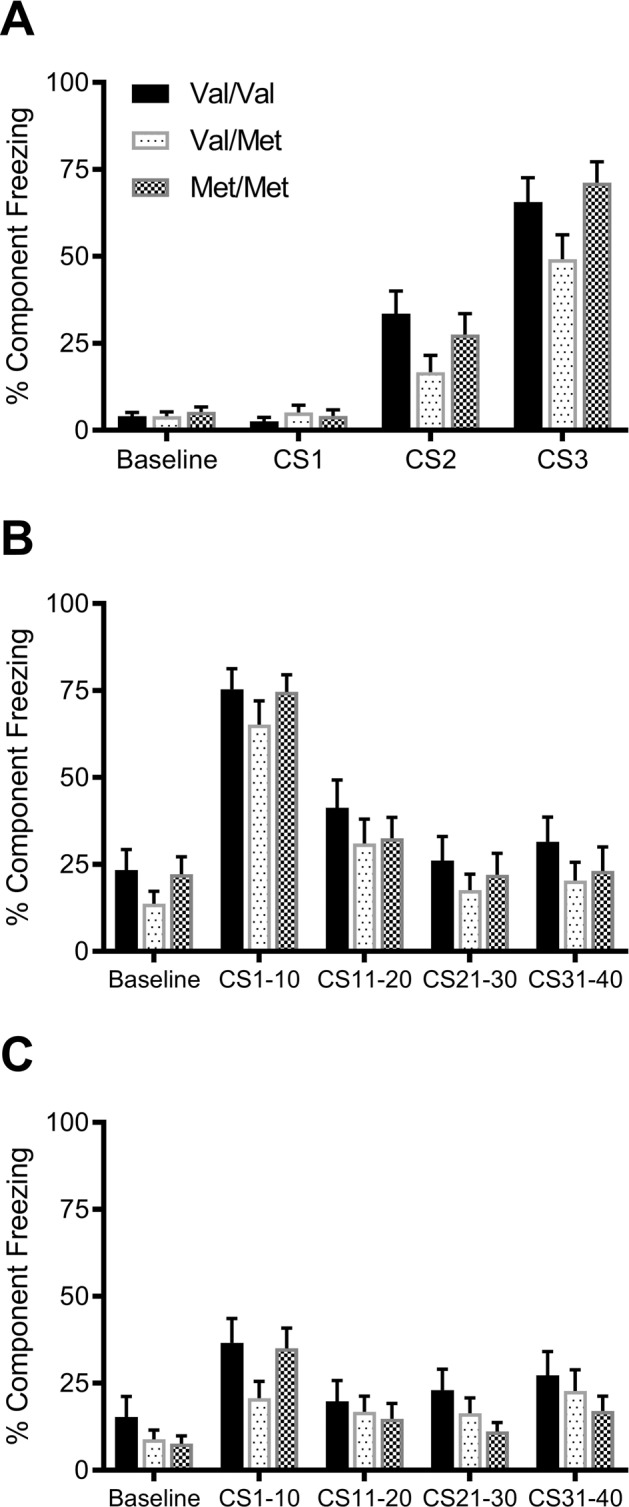
Fear conditioning in adolescent rats. Fear learning across three CS tone periods on Day 1 (**A**), fear memory and extinction learning on Day 2 (**B**) and extinction memory on Day 3 (**C**). There were no significant differences between genotypes at any stage of testing in adolescent rats. Data presented as mean ± SEM. Individual data points for select periods can be found in supplementary Fig. 5. All data are presented as sexes combined.

Fear conditioning was repeated in a separate cohort of Val68Met rats at 4 weeks of age. Analysis of the three CS periods on Day 1 (Fig. 2A) of the fear conditioning protocol showed a large main effect of CS (*F*(2,106) = 132.5, *p* < 0.001, ηp^2^ = 0.71), confirming fear learning, with freezing increasing from CS1 to CS3. There were no main effects of either sex or genotype on fear learning, and no significant interactions between CS and sex (*F*(2,282) = 0.011, *p* = 0.99, ηp^2^ < 0.001) or CS, sex and genotype (*F*(4,282) = 0.71, *p* = 0.57, ηp^2^ = 0.010); however, there was a small interaction between CS and genotype (*F*(4,282) = 0.94, *p* = 0.43, ηp^2^ = 0.013). Further analysis of each CS period separately showed no main effect of genotype during either CS1 (*F*(2,59) = 0.57, *p* = 0.57, ηp^2^ = 0.019), CS2 (*F*(2,59) = 2.13, *p* = 0.13, ηp^2^ = 0.067) or CS3 (*F*(2,59) = 2.79, *p* = 0.070, ηp^2^ = 0.086). These results suggest similar levels of freezing and fear learning in all rats during this session.

Analysis of fear extinction on Day 2 across four groups of 10 CS tones (Fig. 2B) showed a large main effect of CS (*F*(3,159) = 106.1, *p* < 0.001, ηp^2^ = 0.67), showing extinction learning, with freezing decreasing from CS1–10 to CS31–40. There were no significant CS × sex (*F*(3,159) = 1.05, *p* = 0.36, ηp^2^ = 0.019), CS × genotype interaction (*F*(6,159) = 0.26, *p* = 0.92, ηp^2^ = 0.010) or CS × sex × genotype interactions (*F*(6,159) = 0.80, *p* = 0.54, ηp^2^ = 0.029). Along with no significant main effects of sex or genotype, these results show that 4 week old rats of all genotypes showed similar levels of fear memory and extinction learning on Day 2.

Analysis of the four groups of 10 CS tones on Day 3 (Fig. 2C) again shows a large main effect of CS (*F*(3,159) = 13.7, *p* < 0.001, ηp^2^ = 0.21). There was again no significant CS × sex interaction (*F*(3,159) = 0.35, *p* = 0.70, ηp^2^ = 0.007) or a CS × sex × genotype interaction (*F*(6,159) = 2.23, *p* = 0.072, ηp^2^ = 0.078). There was however a medium CS × genotype interaction (*F*(6,159) = 3.16, *p* = 0.018, ηp^2^ = 0.11). Further analysis of each CS block separately showed no main effect of genotype during either CS1–10 (*F*(2,59) = 2.23, *p* = 0.12, ηp^2^ = 0.070), CS11–20 (*F*(2,59) = 0.33, *p* = 0.72, ηp^2^ = 0.011), CS21–30 (*F*(2,59) = 2.21, *p* = 0.12, ηp^2^ = 0.070) or CS 31–40 (*F*(2,59) = 0.94, *p* = 0.34, ηp^2^ = 0.031). These results suggest a similar level of freezing and extinction memory in all young adolescent rats on Day 3.

### No effect of genotype on exploration and anxiety-like behavior in the open field or elevated plus maze

**Fig. 3:**
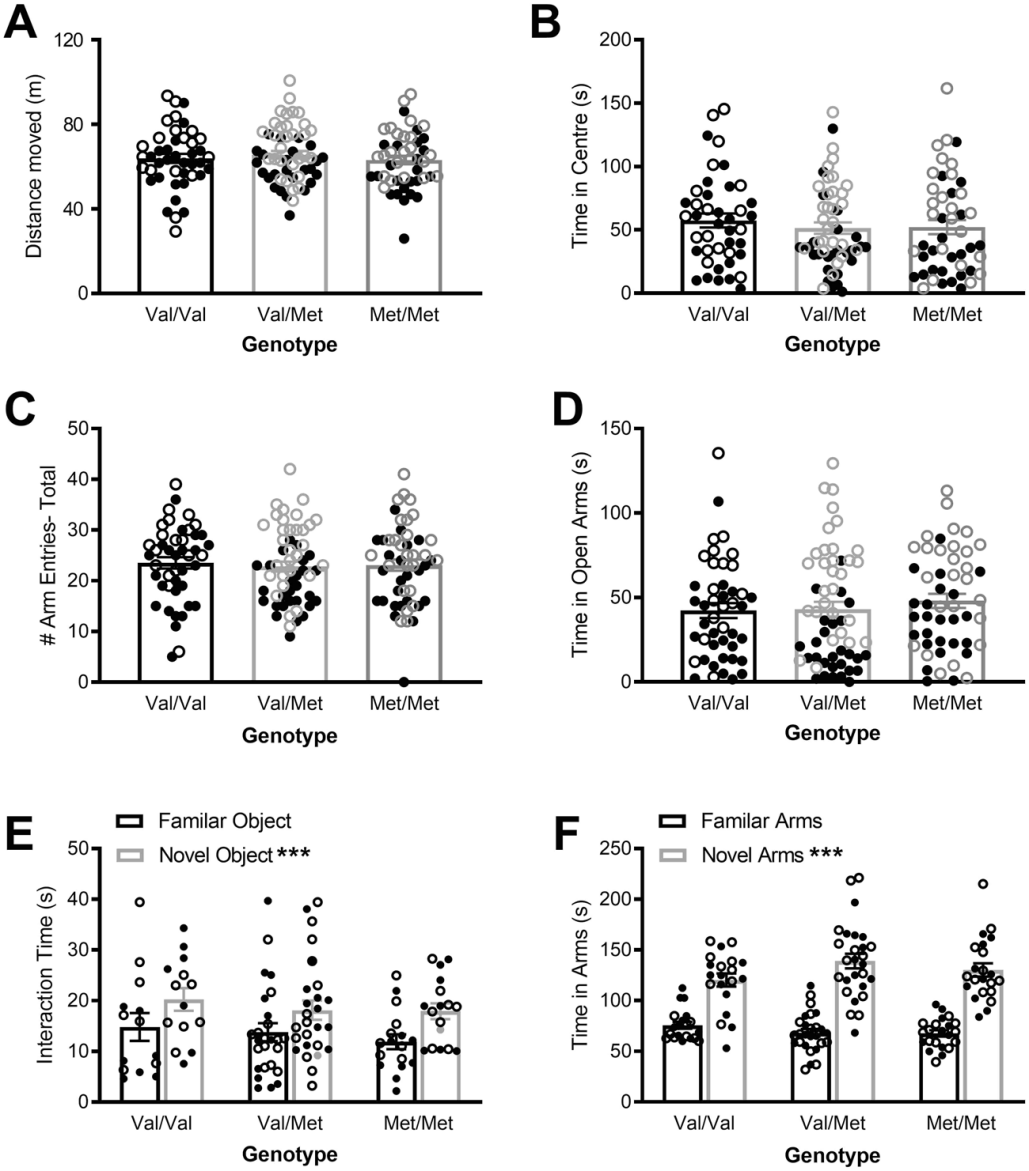
Anxiety-like behavior and learning and memory in adult rats. Distance traveled (**A**) and time spent in the center of the open field (**B**), as well as total number of arm entries (**C**) and time spent in the open arms of the EPM (**D**) demonstrate no differences in baseline locomotor activity or anxiety-like behavior between genotypes. Time interacting with a novel compared to familiar object in the NORT (**E**) and time spent in novel compared to familiar arms of the Y-maze (**F**) showed that, while rats showed a significant preference for the novel object or arm, there were no differences between genotypes. Data presented as mean ± SEM with individual data points for all rats. There were no significant interactions between genotype and sex therefore all data are presented as sexes combined (males closed symbols, females open symbols). ***p < 0.001.

Analysis of distance traveled in the open field (Fig. 3A), used as a measure of baseline locomotor activity, showed a medium main effect of sex (*F*(1,133) = 20.3, *p* < 0.001, ηp^2^ = 0.13), with females being more active than males. However there was no main effect of genotype (*F*(2,133) = 0.51, *p* = 0.60, ηp^2^ = 0.008) or sex × genotype interaction (*F*(2,133) = 0.16, *p* = 0.85, ηp^2^ = 0.002). Similarly, time spent in the center of the open field (Fig. 3B), a measure of anxiety-like behavior, also showed a medium main effect of sex (*F*(1,131) = 17.1, *p* < 0.001, ηp^2^ = 0.12) but not of genotype (*F*(2,131) = 1.03, *p* = 0.36, ηp^2^ = 0.015) or an interaction of these factors (*F*(2,131) = 0.042, *p* = 0.96, ηp^2^ = 0.001). This suggests that female rats have lower levels of anxiety-like behavior compared to males, although this is not affected by Val68Met genotype.

Analysis of time spent in the open arms of the EPM (Fig. 3D), another measure of anxiety-like behavior, showed similar results to the open field. There was a large main effect of sex (*F*(1,140) = 51.0, *p* < 0.001, ηp^2^ = 0.27), with females spending more time in the open arms than males. Again, there was no effect of Val68Met genotype, with no main effect of genotype (*F*(2,140) = 1.13, *p* = 0.33, ηp^2^ = 0.016) or an interaction between sex and genotype (*F*(2,140) = 2.53, *p* = 0.083, ηp^2^ = 0.035). The total number of arm entries were also analyzed as a measure of exploration and activity (Fig. 3C). There was a large main effect of sex (*F*(1,140) = 27.9, *p* < 0.001, ηp^2^ = 0.17), with females more active than males. There was again no effect of Val68Met genotype, with no main effect of genotype (*F*(2,140) = 0.51, *p* = 0.60, ηp^2^ = 0.007) or an interaction between sex and genotype (*F*(2,140) = 0.74, *p* = 0.48, ηp^2^ = 0.010).

There were also no differences in rats tested from 4 weeks of age between the genotypes in terms of either open field or elevated plus maze behavior (Supplementary Fig. 6).

### No effect of genotype on recognition memory in the Novel Object Recognition Test and Y-maze

Analysis of time spent interacting with a novel compared to a familiar object (Fig. 3E) showed a large main effect of object (*F*(1,51) = 15.4, *p* < 0.001, ηp^2^ = 0.23) suggesting an overall preference for the novel object. While there were no object × genotype (*F*(2,51) = 0.17, *p* = 0.85, ηp^2^ = 0.007) or object × genotype × sex interactions (*F*(2,51) = 1.58, *p* = 0.22, ηp^2^ = 0.058), suggesting no role for the Val68Met genotype in recognition memory, there was a significant medium object × sex interaction (*F*(1,51) = 4.50, *p* = 0.039, ηp^2^ = 0.081). Splitting the data by sex shows that, while males demonstrate a large preference for the novel object (*F*(1,25) = 14.3, *p* = 0.001, ηp^2^ = 0.36), females did not (*F*(1,26) = 2.23, *p* = 0.147, ηp^2^ = 0.079).

Analysis of time spent in the novel arm of the Y-maze compared to the average of the two familiar arms (Fig. 3F) showed a large main effect of arm (*F*(1,63) = 99.2, *p* < 0.001, ηp^2^ = 0.61) indicating the expected preference for the novel arm. There were no arm × sex (*F*(1,63) = 1.94, *p* = 0.17, ηp^2^ = 0.030), arm × genotype (*F*(2,63) = 1.41, *p* = 0.25, ηp^2^ = 0.043) or arm × sex × genotype (*F*(2,63) = 0.31, *p* = 0.74, ηp^2^ = 0.010) interactions, suggesting no differences in behavior between sexes, and no role for the Val68Met genotype in spatial recognition memory tested in the Y-maze.

### BDNF mRNA levels in the dorsal hippocampus display increased expression of exon VI in female Val/Met rats

**Fig. 4:**
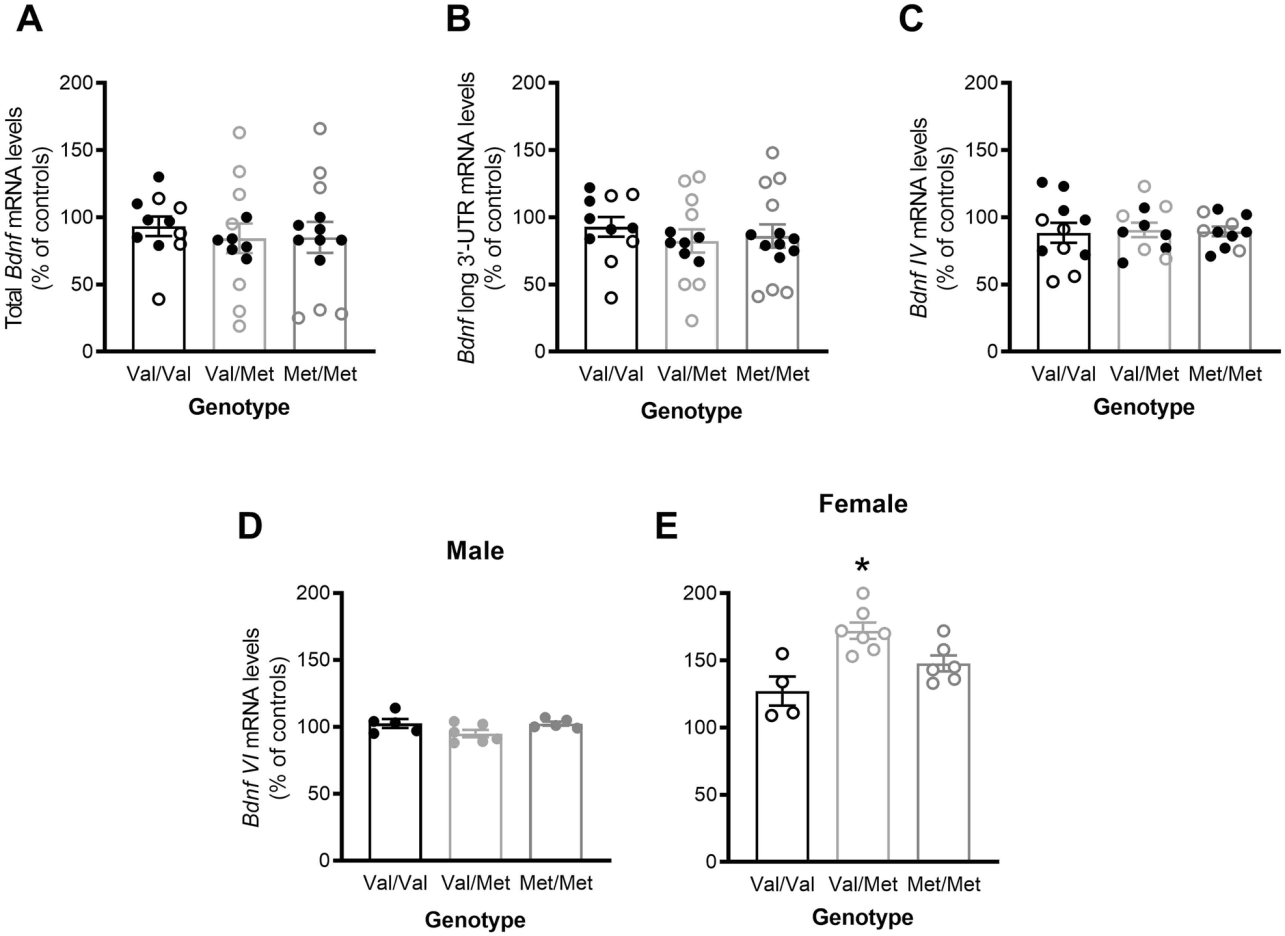
BDNF gene expression in the dorsal hippocampus as measured by RT-qPCR. We measured BDNF total mRNA (**A**), BDNF long 3′ UTR (**B**), BDNF exon IV (**C**) and BDNF exon VI (**D**, **E**) mRNA. Female Val/Met rats showed higher BDNF VI expression than Val/Val controls. Data are presented as % of controls (Male Val/Val rats) showing mean ± SEM of sexes combined with individual data points for all rats (males closed symbols, females open symbols). *p < 0.05 compared to Val/Val rats.

RT-qPCR analysis of total *Bdnf* mRNA levels as well as of some of its isoforms (Fig. 4) revealed no genotype differences for total Bdnf, long 3′-UTR and exon IV (*F*(2,31) = 0.17, *p* = 0.84, ηp² = 0.011; *F*(2,32) = 0.32, *p* = 0.73, ηp² = 0.020; and *F*(2,27) = 0.12, *p* = 0.89, ηp² = 0.009, Fig. 4A, B and C, respectively). Large main effects of genotype (*F*(2,27) = 5.47, *p* = 0.010, ηp² = 0.29) and sex (*F*(1,27) = 114.7, *p* < 0.001, ηp² = 0.81) as well as a genotype × sex interaction (*F*(2,27) = 11.4, *p* < 0.001, ηp² = 0.46) were found for exon VI expression with female rats showing higher mRNA levels than males. Based on the significant sex × genotype interaction, further analysis was done on data from males and females separately. In males (Fig. 4D) there was no effect of genotype (*F*(2,13) = 2.74, *p* = 0.10, ηp² = 0.30) but a large genotype-dependent increase was found in females (*F*(2,14) = 9.34, *p* = 0.003, ηp² = 0.57). Post hoc analysis revealed that Val/Met females had significantly higher BDNF VI expression than Val/Val females (*p* = 0.0023; Fig. 4E).

### BDNF protein levels in the ventral hippocampus are decreased in female Val/Met rats

**Fig. 5:**
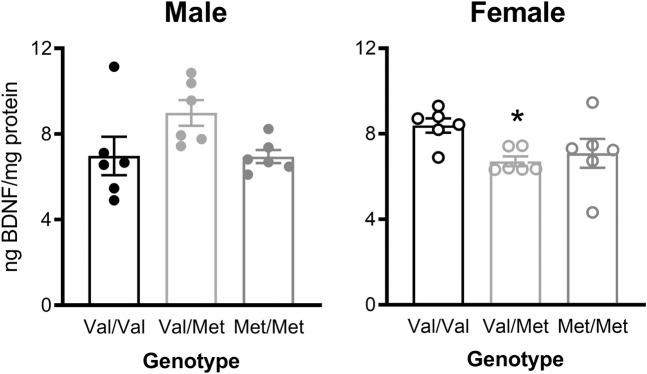
BDNF levels in the ventral hippocampus as measured by ELISA. Female Val/Met rats showed lower BDNF protein levels than Val/Val controls. Data are presented as mean ± SEM with individual data points for all rats. *p < 0.05 compared to Val/Val rats.

Analysis of ventral hippocampal BDNF levels measured by ELISA (Fig. 5) showed no main effects of either genotype (*F*(2,30) = 1.23, *p* = 0.31, ηp^2^ = 0.076) or sex (*F*(1,30) = 0.27, *p* = 0.61, ηp^2^ = 0.009) but there was a large interaction between genotype and sex (*F*(2,30) = 5.60, *p* = 0.009, ηp^2^ = 0.27). Based on this interaction, data were analyzed separately for each sex, with females (*F*(2,15) = 3.75, *p* = 0.048, ηp^2^ = 0.33) but not males (*F*(2,15) = 3.25, *p* = 0.067, ηp^2^ = 0.30) showing a large effect of genotype. Tukey’s multiple comparison post hoc test of female rats showed that Val/Met rats tended to have slightly, but significantly lower BDNF levels than Val/Val (*p* = 0.049; Fig. 5), with no significant differences between other genotypes.

## Discussion

The study investigated the baseline behavioral phenotype of a novel rat model of the Val66Met polymorphism over a range of anxiety, and memory and learning tasks. It was shown that adult rats with the Met/Met genotype demonstrated impaired fear memory compared to Val/Val WT rats in a fear conditioning task, as shown by decreased freezing compared to both Val/Val and Val/Met rats during the 1st ten tones played on Day 2 of the fear conditioning/extinction protocol. Met/Met rats did not show differences in fear learning or extinction. Additionally, the impaired fear memory was seen in the absence of any differences in baseline locomotor activity or anxiety, or other types of recognition memory.

The finding that Met/Met rats display impaired fear memory is consistent with similar findings by others [29, 55] who also found impaired fear memory in mice with the Met/Met genotype, and humans with the 66Met genotype, respectively. Although we did not see any change in other aspects of fear learning and memory associated with the Met allele, other researchers have shown such a link in both rodent and human studies. [29] showed impaired contextual fear memory, which was not measured in the current study, in Met/Met mice conditioned during adolescence and tested as adults, in comparison to the Val/Val genotype, while [55] found that human Met allele carriers demonstrated a deficit in fear conditioning learning. Several studies in humans [30, 32, 56] also found impaired fear extinction learning and memory in Met allele carriers in comparison to the Val/Val genotype, while Soliman et al. [33] have shown normal development of fear conditioning with impaired extinction learning in both mice and humans. Finally, Asthana et al. [57] demonstrated Met carriers with lower reactivation of fear memory following extinction, which we did not measure. Previous studies in a model of the Val66Met mouse carrying a humanized version of the polymorphism [1, 19] used a different protocol and in Met/Met mice saw decreased contextual fear memory, which was not measured in our rats, while [19] also saw a trend for decreased freezing in response to tone presentation, consistent with our finding. However, Chen et al. [1] did not see the same decrease in cue-dependent fear memory. Variations in the laboratories where these tests were conducted may have contributed to these differences. In addition, both studies did not test cued memory until day 3 of the fear conditioning protocol, in contrast to the current study which tested it on day 2 where results may have been stronger, to explain some differences between our results and these mouse studies. Gururajan et al. [22] similarly reported some changes in fear conditioning in BDNF het rats, however at different stages of the protocol than in our Val68Met rats. While there were no differences in fear memory like we observed here, BDNF het rats showed decreased freezing on day 1, or impaired fear learning, and following previous corticosterone treatment, also showed changes in extinction learning and memory. The main difference between our results and other models of Val66Met is that we did not see any differences in extinction as seen by others [33]. One possible explanation for this is that our rats achieved a very high level of extinction, with freezing decreasing to below 10%, which contrasts with levels of above 40% in the model used by these authors [33], which may have masked any small differences that otherwise may have been seen. Future studies could look to use different protocols of fear conditioning, including context memory and different extinction protocols, to investigate the effects of the Met allele further in this rat model. Taken together, the results of our current study as well as previous studies, suggest that BDNF, or BDNF release, is likely to be involved in fear learning, memory and extinction; however, the exact role may differ between the experimental model and/or fear conditioning protocol used.

The two-factor learning theory of fear and anxiety suggests that fear is acquired through classical conditioning processes and is maintained by operant conditioning through the negative reinforcement of avoidance behaviors [58]. The Pavlovian classical conditioning paradigm, which has been utilized to study the acquisition and extinction of fear memories in anxiety disorders [59], provides a link between animal models of fear conditioning and pathologies such as PTSD and phobia in humans. The findings from this study could therefore provide evidence of a possible protective effect of the Met allele in the development of affective pathologies such as PTSD and phobia [6]. The reduced fear memory did not represent a generalized cognitive deficit because there were no differences between the genotypes in the Y-maze and NORT tasks and all genotypes showed the expected preference for both the novel object in the NORT and the novel arm in the Y-maze. In contrast to our Y-maze findings, in previous studies [19, 22] we found showed decreased novel arm preference in the Y-maze in Val66Met mice and BDNF het rats. These data leave open the possibility that the Val66Met genotype is associated with deficits in some aspects of learning and memory other than fear memory although not in this novel Val68Met rat model.

The differences in fear memory between Met/Met and Val/Val rats are unlikely to be due to altered baseline locomotor activity or anxiety. Although female rats showed higher activity and lower anxiety-like behavior in the EPM and open field, as well as overall decreased freezing on Day 2 and 3 of the fear conditioning task, no such differences were observed between genotypes, nor any significant interactions between sex and genotype. Although human meta-analytic studies have shown that individuals homozygous for the Met allele have an increased risk for developing anxiety disorders [10, 16, 18], and Chen et al. [1] found an anxiety-like phenotype in Met/Met mice compared to Val/Val controls, our previous studies in mice showed no differences in genotypes in light-dark box measure of anxiety [19], while BDNF het rats showed an anxiety phenotype in the open field but not EPM [21]. Interestingly, previous studies in a Val66Met mouse model have shown impaired spatial memory in Met/Met females but not males [60], as well fluctuations in anxiety-like behavior and spatial memory across the estrous cycle in Met/Met mice [61, 62]. Although we did not see any sex by genotype interactions in our rats, future studies could investigate behaviors at different stages of the estrous cycle to confirm this lack of genotype difference between sexes.

Several previous studies have shown that adolescent rats behave differently in fear conditioning protocols than adult rats [38–40], with juvenile and adult rats showing a higher degree of fear extinction than adolescent rats [38]. Moreover, we have shown in mice that the developmental profile of BDNF expression markedly changes during the transition from juvenile age to adulthood [63]. Specifically, male mice showed an age-dependent peak of forebrain BDNF expression which was associated with a surge in testosterone in the serum. Female mice showed an earlier and more gradual rise in BDNF signaling during adolescent development and these appeared not directly associated with serum estrogen levels [63]. These observations are yet to be repeated in mice or rats with the Val68Met SNP. The present study shows that the difference in fear memory in adult Met/Met rats was not seen at 4 weeks of age during the early stages of adolescence, suggesting a possible developmental component to the results seen.

In contrast to the behavioral results, gene expression analyses revealed some sex-specific genotype differences. While there were no baseline differences between the groups in total Bdnf, long 3′-UTR and exon IV mRNA levels, overall, female rats presented with higher levels of exon VI mRNA than males, with Val/Met females specifically showing higher levels than Val/Val females. It is well known that BDNF gene expression may be influenced by estrogen [64] including in the hippocampus [65]. The BDNF Val66Met polymorphism has also been linked to sex differences in major depressive disorders and stress reactivity in humans [66]. Previous work has furthermore shown that Met/Met male mice have altered expression of Bdnf total, 3′-UTR and exon IV in the whole hippocampus [46, 47] although we did not find any significant changes of these isoforms when investigating the dorsal hippocampus of male and female Val68Met rats. A significant reduction of BDNF VI expression was also found in Met/Met mice [47], which would appear opposite to our finding of increased BDNF VI expression in Val/Met rats. However, the previous studies [47] included only male mice and our finding of increased BDNF VI expression was selective for female Val/Met rats, suggesting that differences in the gene expression effects of Val66Met may be both sex- and species-specific. Studies in female Val66Met mice looking at in situ hybridization expression of BDNF and TrkB in the CA3 and CA1 regions of the hippocampus respectively showed increased expression of both factors in Met/Met mice [62]. It will be interesting to see if future studies involving different interventions display further sex differences in these measure in our rats. It is interesting that a change in expression of any of the genes measured was seen in Val/Met rats, and not the more extreme Met/Met genotype. It is possible that the homozygous genetic modification (in this case Met/Met) is more likely to trigger compensatory mechanisms to overcome any downstream changes induced by the decreased BDNF release at the synapse and therefore appears to show no effect. In contrast, the less robust BDNF release deficit in the heterozygous Val/Met animals may not have induced such compensatory mechanisms [67] and shows a functional deficit. Previously we have seen a similar effect of genotype on prepulse inhibition in the Val66Met mouse model where Val/Met mice showed significantly reduced PPI but Met/Met mice did not [20]. It should also be noted that many previous papers in Val66Met mouse models have only studied Val/Val and Met/Met genotypes, which could have missed possible differences in the heterozygous Val/Met animals. The functional consequence of increased BDNF VI expression in the dorsal hippocampus could be higher BDNF translation specifically in distal dendrites as opposed to somatic and proximal dendrites [44]. However, the increase of exon VI in Val/Met females only may be unrelated to the deficit in fear memory which was seen in both male and female rats of the Met/Met genotype only.

ELISA showed no differences between the genotypes in the levels of BDNF in the ventral hippocampus when sexes were combined, although a small decrease was observed in Val/Met females. We previously observed a similar result of no change in BDNF protein levels in the dorsal hippocampus, ventral hippocampus, and medial prefrontal cortex of Val66Met mice [20], in line with a previous study reporting that BDNF release, not BDNF levels, were reduced in rats with the Met/Met genotype [34]. There were also no significant differences between genotypes in full-length, truncated, or phosphorylated TrkB expression in Val66Met mice [20] but further studies are required to verify if this is similarly the case in our Val68Met rat model. In addition to BDNF levels, brain weights were measured (Supplementary Results) and no differences in genotype were seen, either as gross weight or as a percentage of body weight.

In conclusion, the Met/Met genotype of the Val68Met rat model shows impaired fear memory which is not associated with any other differences in fear learning or extinction or other forms of learning and memory. This impairment is also not associated with any other baseline differences in locomotor activity or anxiety, and may suggest a specific developmental impairment in specific regions of the brain. It is important to note that some of these findings are different from results shown in various mouse models of the Val66Met polymorphism engineered in different ways and generated in different laboratories. Examples included in this discussion have used a humanized version of the Val66Met gene [1], which is different from our rat model where the BDNF gene was altered at the equivalent position in rodents [34]. Along with protocol differences and variations in laboratory environments breeding, housing and experiments are conducted in, this may contribute to some of the behavioral differences seen between studies. Rats have been suggested to show advantages over mice due to the rich and complex behavioral repertoire of the rat, for example in drug abuse-related paradigms [36]. As the Val66Met polymorphism has been suggested as a putative locus of risk for anxiety and affective disorders [1, 2, 6], and, importantly, literature implicates the BDNF66Met allele as a marker of substance use disorder susceptibility [68, 69], it will be important to extend the findings of mouse studies in rats as well. The current findings provide a baseline from which gene–environment studies can be undertaken in this animal model to further characterize the role of the BDNF Val66Met polymorphism in health and disease.

## Supplementary information

Supplementary files

## Data Availability

Raw data are available from the authors upon request. BDNF rs6265 Met/Met breeding pairs can be shared by request to Caryl E. Sortwell, Ph.D., Michigan State University, sortwell@msu.edu.
