# Supplementary material for: Behavioral phenotyping of a rat model of the BDNF Val66Met polymorphism reveals selective impairment of fear memory
Source: Transl Psychiatry. 2022 Mar 7;12:93. doi: 10.1038/s41398-022-01858-5 (PMC8901920; doi:10.1038/s41398-022-01858-5)
Supplement: Supplementary file 1 — Supplementary files [file 41398_2022_1858_MOESM1_ESM.docx]

**- Translational Psychiatry -**

**Behavioural phenotyping of a rat model of the BDNF Val66Met polymorphism reveals selective impairment of fear memory**

Emily J. Jaehne^1*^, Jessica N. Kent^1^, Emily J. Antolasic^1^, Bradley J. Wright^1^, Jereme G. Spiers^2^, Kerstin C. Creutzberg^3^, Federico De Rosa^3^, Marco A. Riva^3,4^, Caryl E. Sortwell^5,6^, Timothy J. Collier^5,6^, Maarten van den Buuse^1,7,8^

1. Department of Psychology and Counselling, School of Psychology and Public Health, La Trobe University, Melbourne, Australia

2. Department of Biochemistry and Genetics, La Trobe Institute for Molecular Science, La Trobe University, Melbourne, Australia

3. Department of Pharmacological and Biomolecular Sciences, University of Milan, Milan, Italy

4. Biological Psychiatry Laboratory, IRCCS Istituto Centro San Giovanni di Dio Fatebenefratelli, Brescia, Italy

5. Department of Translational Neuroscience, College of Human Medicine, Michigan State University, Grand Rapids, USA

6. Hauenstein Neuroscience Center, Mercy Health Saint Mary's, Grand Rapids, USA

7. Department of Pharmacology, University of Melbourne, Melbourne, Australia

8. College of Public Health, Medical and Veterinary Sciences, James Cook University, Townsville, Australia

**Supplementary Results**

**Supplementary Table 1**

Breeding colony details showing number of pups born over the course of data collection for this project, including animals used in other projects and excess stock produced. Data are shown split by genotype for males and females combined and for males and females separately. Genotype distribution shows approximately Mendelian distribution.

| Genotype | Number | % Total |  |
| --- | --- | --- | --- |
| Val/Val | 123 | 24.3 |  |
| Val/Met | 252 | 49.8 |  |
| Met/Met | 130 | 25.7 |  |
| Total | 506 |  |  |
|  |  |  | % per sex |
| Val/Val male | 71 | 14.0 | 27.8 |
| Val/Met male | 124 | 24.5 | 48.6 |
| Met/Met male | 60 | 11.9 | 23.5 |
| Val/Val female | 52 | 10.3 | 20.7 |
| Val/Met female | 128 | 25.3 | 51.0 |
| Met/Met female | 70 | 13.8 | 27.9 |
| Total male | 255 |  |  |
| Total female | 251 |  |  |

**Supplementary Table 2**: number of animals per group.

|  | **Genotype** | | |
| --- | --- | --- | --- |
|  | **Val/Val** | **Val/Met** | **Met/Met** |
| **Cohort 1** |  |  |  |
| Male | 10 | 12 | 10 |
| Female | 7 | 14 | 12 |
| **Cohort 2** |  |  |  |
| Male | 11 | 13 | 10 |
| Female | 9 | 14 | 12 |
| **Cohort 3** |  |  |  |
| Male | 16 | 15 | 15 |
| Female | 12 | 16 | 14 |
| **4-Week Old Cohort** |  |  |  |
| Male | 10 | 11 | 9 |
| Female | 11 | 11 | 13 |

**Supplementary Figure 1: Body Weight**


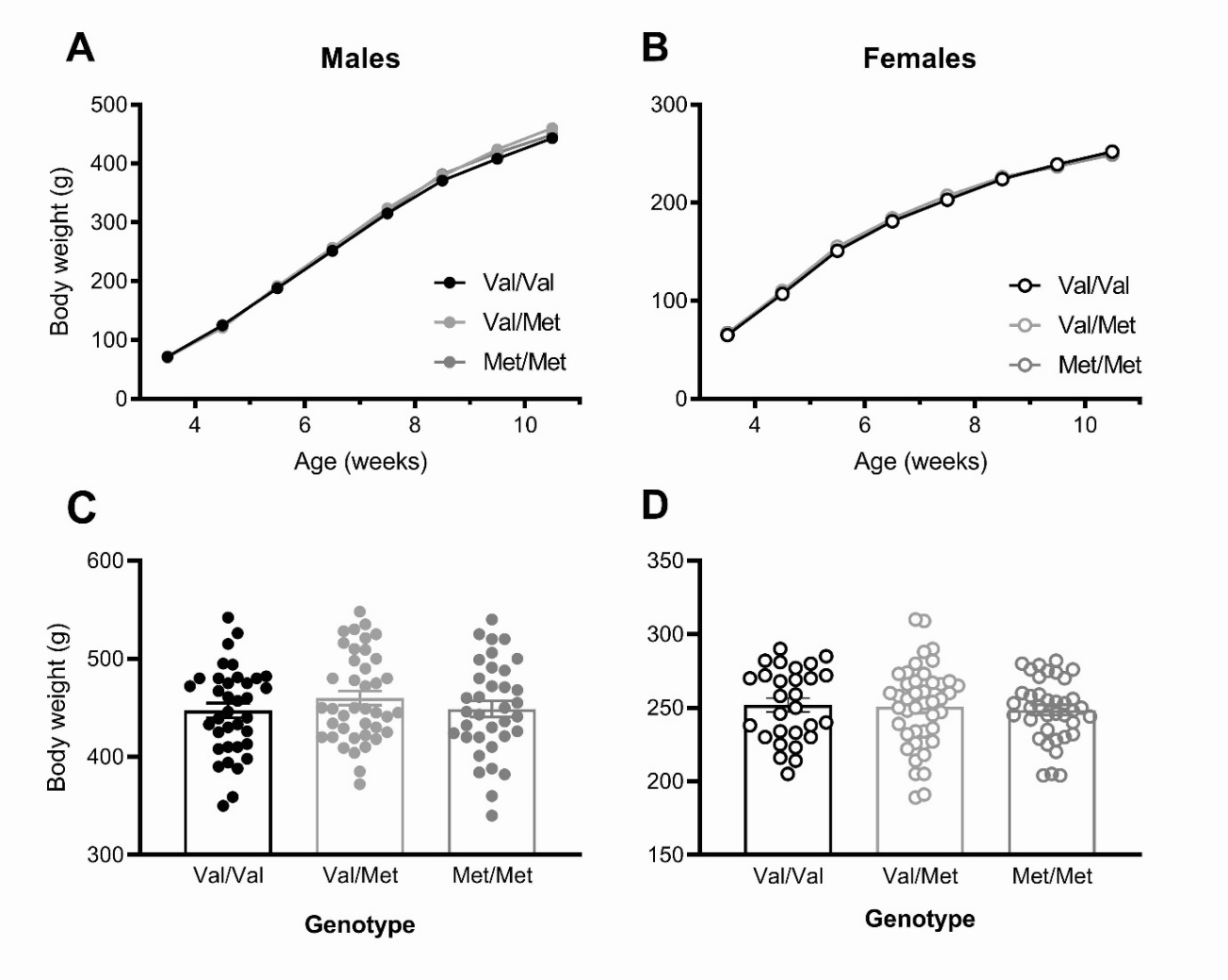


**Supplementary Figure 1**. Body weight of male (A) and female (B) rats over time from weaning at 3 weeks of age until the end of behavioural testing at 10-11 weeks of age and final body weight collected of male (C) and female (D) rats. Data represent mean ± SEM with individual data points shown for final body weight only.

All rats were weighed from weaning at 3 weeks of age until the end of behavioural testing at 10-11 weeks of age. Given the obvious weight difference between male and female rats, data have been analyzed separately for each sex. Repeated measures ANOVA of male rat body weights showed a significant main effect of age (F(7,742) = 6527, p < 0.001, ηp^2^ = 0.989) but not a main effect of genotype (F(2,106) = 0.42, p = 0.66, ηp^2^ = 0.008) or an interaction of genotype x age (F(14,742) = 2.05, p = 0.097, ηp^2^ = 0.104). Similarly for female rats, analysis showed a significant main effect of age (F(7,728) = 5007, p < 0.001, ηp^2^ = 0.991) but not a main effect of genotype (F(2,104) = 0.11, p = 0.90, ηp^2^ = 0.002) or an interaction of genotype x age (F(14,728) = 0.71, p = 0.62, ηp^2^ = 0.076). These results show that there are no genotype effects on body weight over the ages measured.

**Supplementary Figure 2: Brain weights**


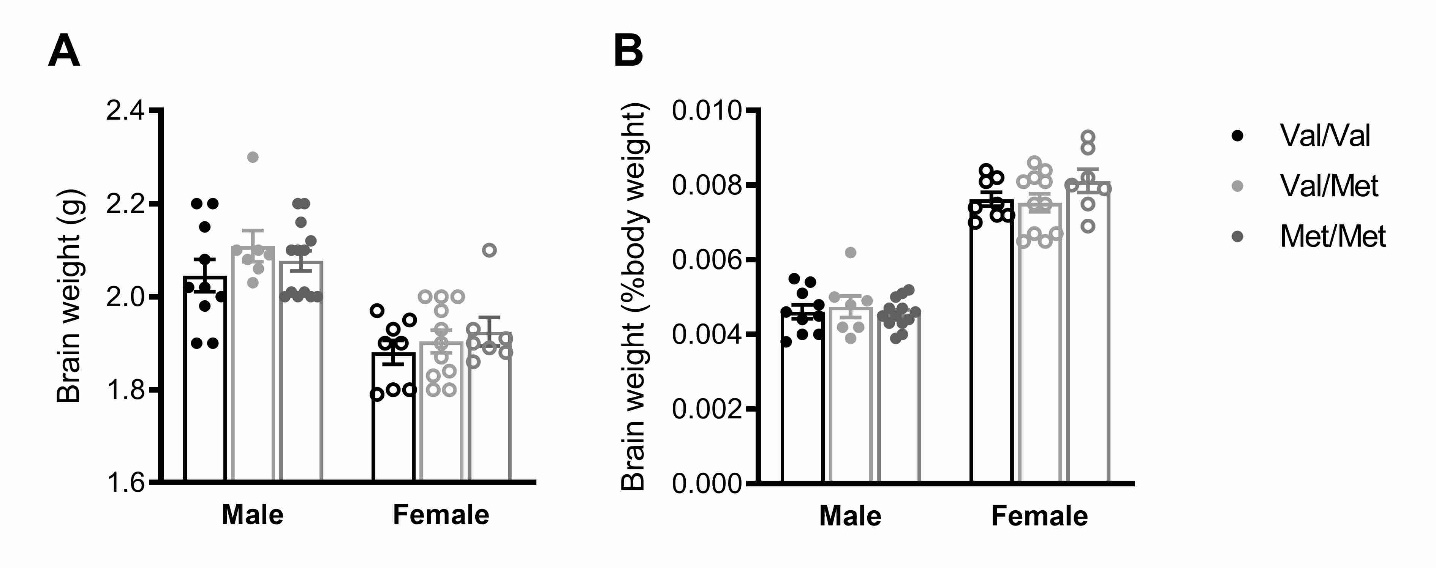


**Supplementary Figure 2.** Brain weights expressed as actual weight (A) and as a percentage of final body weight (B). Data represent mean ± SEM with individual data points.

A random selection of brains were weighed (male VV n = 10, male VM n = 7, male MM n = 13, female VV n = 8, female VM n = 11, female MM n = 7) at tissue collection. Analysis of brain weight using 2-way ANOVA showed a significant main effect of sex (F(1,48) = 53.4, p < 0.001, ηp^2^ = 0.53), with females having smaller brains than males, but no main effect of genotype (F(2,48) = 1.30, p = 0.28, ηp^2^ = 0.051) or an interaction of the two factors (F(2,48) = 0.33, p = 0.72, ηp^2^ = 0.013). Similarly, when brain weight as a percentage of body weight was analyzed, there was again a main effect of sex (F(1,48) = 289.2, p < 0.001, ηp^2^ = 0.86) with females showing higher brain size as a percentage of body weight, but no main effect of genotype (F(2,48) = 0.52, p = 0.60, ηp^2^ = 0.021) or sex x genotype interaction (F(2,48) = 1.63, p = 0.21, ηp^2^ = 0.063). These results show that val68met genotype has no effect on brain weight of rats.

**Supplementary Figure 3: Fear conditioning Receiver Operating Characteristic (ROC) curve analysis**


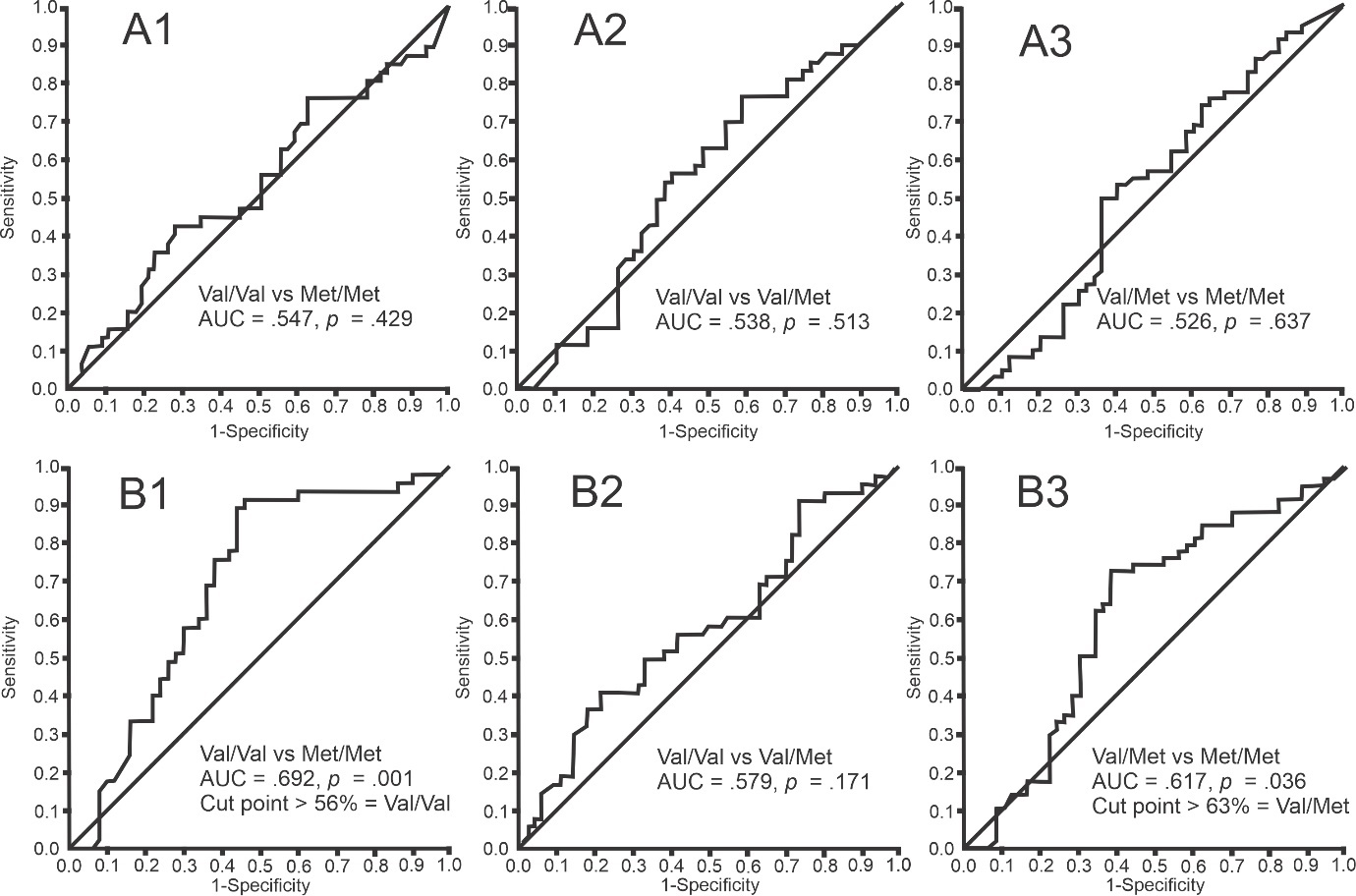


**Supplementary Figure 3.** Receiver operating characteristic (ROC) curves, corresponding area under the curve (AUC), sensitivity, and specificity of freezing behavior for distinguishing genotype. Panels A1-3 represent freezing behavior on the extinction learning task. Panels B1-3 represent freezing behavior on the fear memory task. Panel B1 illustrates that higher freezing behavior discriminates Val/Val rats from Met/Met rats, with a cut-point of 56% correctly classifying 91% of Val/Val rats.

**Supplementary Figure 4: NORT Training Phase**

**Supplementary Figure 4.** Time interacting with a both objects in total (A) and time spent with the left and right object (B) during the training phase of the NORT. While females rats explored the objects more, there was no difference between genotypes in total time spent exploring objects. There was a slight, but significant effect of object side, with a preference for the right object, however there was no interaction of this with either genotype or sex. Data presented as mean ± SEM with individual data points for all rats. There were no significant interactions between genotype and sex therefore all data are presented as sexes combined (males closed symbols, females open symbols). * p < 0.05.

**NORT Training**

Analysis of total time spent interacting with both objects during the training phase of the NORT showed a significant effect of sex (F(1,56) = 5.56, p = 0.022, ηp^2^ = 0.090), with females exploring the objects more than males, but no significant effect of genotype (F(2,56) = 0.42, p = 0.66, ηp^2^ = 0.015). Comparison of time spent with the left object compared to the right object during this phase did show a significant effect of object (F(1,56) = 6.40, p = 0.014, ηp^2^ = 0.10), with rats spending 33.3 ± 2.29 s with the right object and only 29.0 ± 1.85 s with the left. As the side the novel object was placed in the testing phase was always randomized between the two sides of the chamber, we believe this small preference would not have had an effect on the results of the testing phase.

**Supplementary Figure 5:** **Behavioural results 4 week old rats - Fear conditioning**


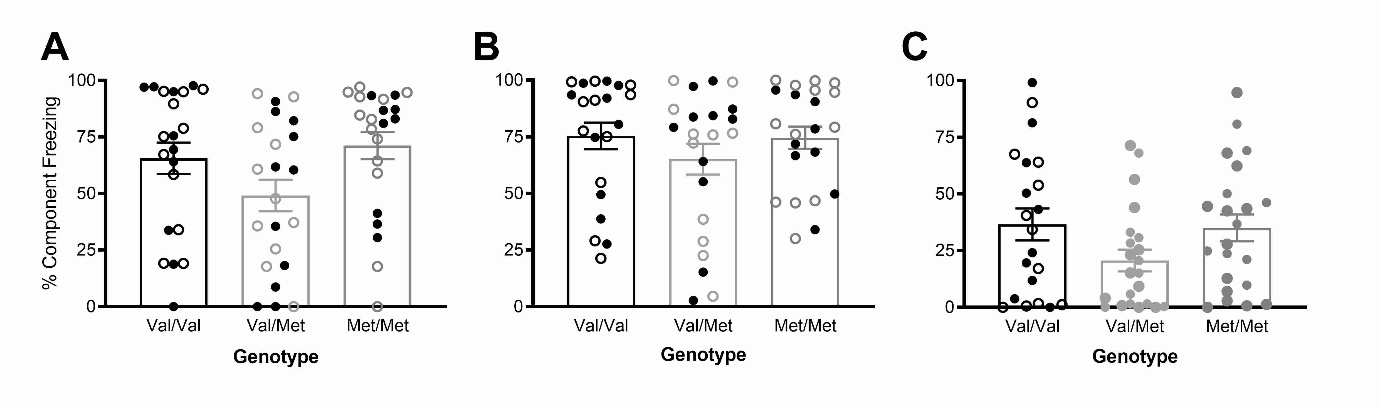


**Supplementary Figure 5.** Fear learning in adolescence across on Day 1 CS3 (A), fear memory during Day 2 CS1-10 (B), and extinction recall during Day 3 CS1-10 (C). There were no significant differences between genotypes at any stage of testing in 4 week old rats. Data are presented as mean ± SEM with individual data points shown. There were no significant interactions with sex therefore all data are presented as sexes combined (males closed symbols, females open symbols). For detailed statistical analysis, see main text.

**Supplementary Figure 6: Behavioural results 4 week old rats - Open field and Elevated plus maze**


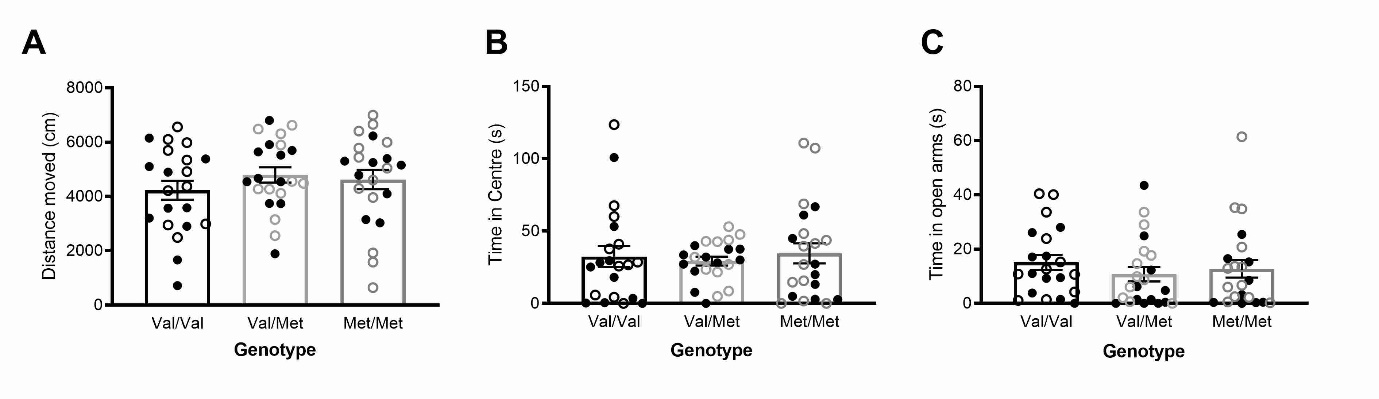


**Supplementary Figure 6.** Analysis of distance travelled in the open field (A), time spent in the center of the open field (B) and time spent in the open arms of the EPM (C) showed no differences in baseline locomotor activity or anxiety-like behavior between genotypes. Rats started behavioural testing at 4 weeks of age. Data are presented as mean ± SEM with individual data points for all rats. There were no significant interactions between genotype and sex therefore all data are presented as sexes combined (males closed symbols, females open symbols).

**Open Field**

Analysis of distance travelled in the open field (Supplementary Figure 5A), used as a measure of baseline locomotor activity, showed no significant main effect of sex (F(1,59) = 0.51, p = 0.48, ηp^2^ = 0.009) or genotype (F(2,59) = 0.82, p = 0.45, ηp^2^ = 0.027) and no or sex x genotype interaction (F(2,59) = 0.82, p = 0.45, ηp^2^ = 0.027). Similarly, time spent in the center of the open field (Supplementary Figure 5B), a measure of anxiety-like behavior, also showed no significant main effect of sex (F(1,59) = 1.99, p = 0.16, ηp^2^ = 0.033) or genotype (F(2,59) = 0.13, p = 0.88, ηp^2^ = 0.004), and no interaction of the factors (F(2,59) = 0.14, p = 0.87, ηp^2^ = 0.005).

**Elevated Plus Maze**

Analysis of time spent in the open arms of the EPM (Supplementary Figure 5C), another measure of anxiety-like behavior, showed similar results to the open field. There was no main effect of sex (F(1,59) = 3.15, p = 0.081, ηp^2^ = 0.051), and again, there was no effect of Val68Met genotype, with no main effect of genotype (F(2,59) = 0.56, p = 0.57, ηp^2^ = 0.019) or an interaction between sex and genotype (F(2,59) = 0.14, p = 0.87, ηp^2^ = 0.005).
